# Elevated serum procollagen type III peptide in splanchnic and peripheral circulation of patients with inflammatory bowel disease submitted to surgery

**DOI:** 10.1186/1471-230X-4-29

**Published:** 2004-11-04

**Authors:** Matilde De Simone, Ugo Cioffi, Ettore Contessini-Avesani, Barbara Oreggia, Roberta Paliotti, Alberto Pierini, Gianni Bolla, Elide Oggiano, Stefano Ferrero, Fabio Magrini, Michele M Ciulla

**Affiliations:** 1 Department of Surgery, Ospedale Maggiore di Milano, IRCCS, University of Milan, V. F. Sforza, 35 – 20122, Milan, Italy; 2 Istituto di Medicina Cardiovascolare, Centro Interuniversitario di Fisiologia Clinica e Ipertensione, Ospedale Maggiore di Milano, IRCCS, University of Milan, V. F. Sforza, 35 – 20122, Milan, Italy; 3 II Cattedra di Anatomia Patologica, Dipartimento di Medicina Chirurgia e Odontoiatria, A.O. San Paolo and Ospedale Maggiore di Milano, IRCCS, University of Milan, V. A. di Rudinì – 20100, Milan, Italy

## Abstract

**Background:**

In the hypothesis that the increased collagen metabolism in the intestinal wall of patients affected by inflammatory bowel disease (IBD) is reflected in the systemic circulation, we aimed the study to evaluate serum level of procollagen III peptide (PIIIP) in peripheral and splanchnic circulation by a commercial radioimmunoassay of patients with different histories of disease.

**Methods:**

Twenty-seven patients, 17 with Crohn and 10 with ulcerative colitis submitted to surgery were studied. Blood samples were obtained before surgery from a peripheral vein and during surgery from the mesenteric vein draining the affected intestinal segment. Fifteen healthy age and sex matched subjects were studied to determine normal range for peripheral PIIIP.

**Results:**

In IBD patients peripheral PIIIP level was significantly higher if compared with controls (5.0 ± 1.9 vs 2.7 ± 0.7 μg/l; p = 0.0001); splanchnic PIIIP level was 5.5 ± 2.6 μg/l showing a positive gradient between splanchnic and peripheral concentrations of PIIIP. No significant differences between groups nor correlations with patients' age and duration of disease were found.

**Conclusions:**

We provide evidence that the increased local collagen metabolism in active IBD is reflected also in the systemic circulation irrespective of the history of the disease, suggesting that PIIIP should be considered more appropiately as a marker of the activity phases of IBD.

## Background

Crohn's disease (CD) and Ulcerative Colitis (UC) are chronic inflammatory bowel diseases (IBD) of unknown origin of adolescent and young adulthood [1] where genetic polimorphisms [2,3], abnormal inflammation pathways activation [4], and environmental influences [5] seem to concur at different levels in the pathogenesis and the progression of IBD. These pathologic conditions are characterized by focal or diffuse inflammation of the alimentary tract, mucosal damage and epithelial destruction. IBD may be associated with an inability of the intestinal mucosa to protect itself from luminal challenges and inappropriate repair following intestinal injury [6-10]. CD differs from UC by the transmural granulomatous inflammation generally leading to fibrosis, strictures and fistulas [11].

Current opinions suggest that an increased synthesis of collagen type I, III, and V may play an important role in the pathophysiological mechanism leading to intestinal fibrosis [12-15]. An increased synthesis of collagen, namely an increased of procollagen type III, is well documented in fibrotic processes involving other organs such as liver, pancreas, and lung [16-18].

However, not all authors are in agreement regarding the increased serum levels of the aminoterminal propeptide (PIIIP) of collagen in peripheral and splanchnic circulation of patients with active IBD [13,14]. Below we present the results on the serum level of PIIIP in splanchnic and peripheral circulation in patients with active IBD submitted to surgery.

## Methods

Twenty-seven patients affected by active IBD, 17 with CD (age 40.2 ± 13.1, yrs from diagnosis 9.2 ± 5.5), and 10 with UC (age 50.3 ± 15.6, yrs from diagnosis 9.8 ± 7.4) submitted to surgery, were enrolled in the study in a double blind fashion. The protocol was approved by local Ethical Committee and informed consent was obtained from all participants to the study. Three patients had CD in small bowel only, two in large bowel only, 12 had ileocolonic disease. Disease activity was assessed according to the Crohn's Disease Activity Index (CDAI) [19] and the Truelove-Witts index (TWI) [20] for CD and UC, respectively. According to CDAI, 2 patients were subclassified as having a moderate form of disease while 15 patients were subclassified as having a severe form of disease. According to TWI, 3 patients were classified as having moderate form of disease, 4 a mild form, and 3 a severe form of disease.

Patients affected by CD were operated on for recurrent obstruction, whereas patients with UC were submitted to surgery because of refractory to medical therapy.

**Figure 1:**
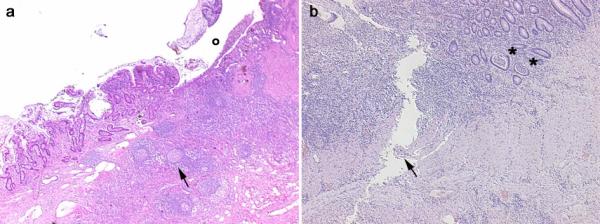
Histological images obtained from two IBD patients enrolled in the study affected by CD (panel a) and UC (panel b) with 12.0 and 10.3 μg/l splanchnic levels of PIIIP, respectively. *Panel a*, CD: in the transmural section is clearly evident an ulceration (o) in the mucosa and submucosa with diffuse inflammatory infiltrations, pseudo-follicle nodules (arrow), and fibrosis of the intestinal wall. *Panel b, UC: *the inflammatory infiltration is more evident in the mucosa and submucosa with criptic abscesses (asterisks). A serpiginous linear ulcer is evident (arrow).

The clinical diagnosis was confirmed by histology (Fig. 1); all cases under study fulfilled the histological criteria as follows:

- for CD: deep ulcers, marked proliferation of small lymphoid nodules involving all layers of intestinal wall sometime with sarcoid-type granulomas and serosal inflammation;

- for UC: mucosal erosions and superficial ulcerations usually limited to the upper submucosa with cryptic abscesses and glandular destruction.

The clinical profile of the studied patients is reported (Table 1). Two patients did not receive any medication, whereas other patients received two or three drugs for the treatment of IBD. Table 2 shows the treatment protocol for all the studied patients. A control group of 15 healthy age and gender matched subjects was also studied to determine normal range for peripheral PIIIP.

**Table 1:** Clinical aspects of the studied patients

**Disease**	**N° of patients**	**Age**	**Sex**	**Years from diagnosis**	**Activity index**
**Crohn**					**CDAI**
	1	34	F	12	Severe
	2	35	M	8	Moderate
	3	38	M	15	Severe
	4	32	F	8	Severe
	5	43	F	14	Severe
	6	35	M	8	Severe
	7	58	M	5	Severe
	8	61	F	7	Severe
	9	33	M	8	Severe
	10	35	F	19	Severe
	11	21	F	4	Severe
	12	72	F	1	Severe
	13	36	M	15	Moderate
	14	42	M	7	Severe
	15	25	F	6	Severe
	16	51	F	18	Severe
	17	34	M	1	Severe
**Ulcerative colitis**					**TWI**
	1	68	F	15	Severe
	2	70	F	1	Moderate
	3	21	F	3	Mild
	4	54	F	20	Moderate
	5	52	M	20	Mild
	6	38	M	4	Moderate
	7	64	M	6	Mild
	8	38	F	16	Severe
	9	41	F	3	Severe
	10	57	M	10	Mild

CDAI: Crohn's Disease Activity Index; TWI: truelove-Witts index.

**Table 2:** Frequency distribution for therapy

**Therapy**	**N° of patients**	**Crohn**	**Ulcerative colitis**
No	2	1	1
Aminosalicydic acid	9	8	1
Cortisone	2	2	0
Aminosalicydic acid + Cortisone	14	6	8
Total	27	17	10

### Collagen metabolism (PIIIP)

Different kinds of collagen have been identified in humans. All of them derive from longer precursor molecules (procollagens). They are synthesized intracellularly and secreted in extracellular space where they are cleaved by aminoproteases [21-23]. Among the different kinds of precursors, type III is one of the most abundant interstitial procollagens. Since its aminoterminal propeptide, PIIIP, is formed in equimolar proportions to collagen, serum measurements of this fragment can provide an index of collagen synthesis [23]. The blood samples (two, 5-ml each) for PIIIP measurements were taken from the median cubital vein (p-PIIIP) before surgery after an overnight fast, during surgery from a mesenteric vein (s-PIIIP) draining the intestinal segment chosen for resection by the surgeon. Serum levels of PIIIP were assessed by commercial radioimmunoassay (Orion Diagnostics, Finland). The intra-assay and inter-assay variation were respectively 4% and 4.3%, mean 2.6 μg/l. Normal ranges of peripheral PIIIP concentrations assessed in the control group were 2.7 ± 0.7 μg/l.

### Statistical analysis

Data were analyzed using a computer statistical software (SPSS-Rel 10; SPSS Inc., Chicago, Ill). All the quantitative variables were tested for Gaussian distribution with the Kolmogorov-Smirnov test. All that followed this distribution were presented as mean ± standard deviation.

Differences at baseline in collagen parameters between IBD patients and controls were tested for significance using the analysis of variance with the Bonferroni correction. The relation between collagen parameters and the estimated duration of the disease and indices of disease were tested with regression analysis. In all cases, a p value less than 0.05 was considered significant.

## Results

### Peripheral PIIIP assay

**Figure 2:**
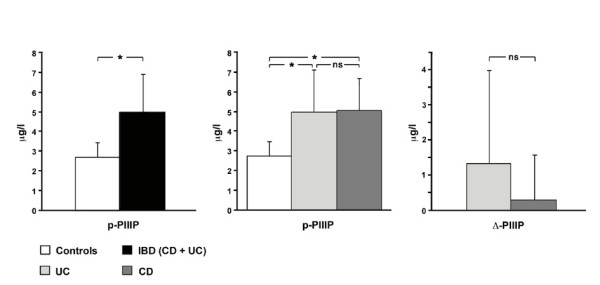
*Panel a: *Differences in baseline p-PIIIP values in Controls and IBD patients. *Panel b: *No significant differences in p-PIIIP values between CD and UC subgroups. *Panel c: *Differences between splancnic and periferic values of PIIIP, without significant differences in CD and UC sbgroups. p-PIIIP: periferic (median cubital vein) PIIIP; s-PIIIP: splancnic (mesenteric vein) PIIIP; Δ-PIIIP: differences between s- and p-PIIIP in IBD patients; IBD: inflammatory bowel diseases; CD: Crohn's Disease; UC: ulcerative colitis;

At baseline, before surgery, serum p-PIIIP in IBD patients were significantly higher if compared with healthy controls (5.0 ± 1.9 vs 2.7 ± 0.7 μg/l, respectively; p = 0.0001) (fig 2a). No significant differences were found when comparing CD and UC subgroups (5.0 ± 1.6 vs 4.9 ± 2.4 μg/l, respectively; p = ns) (fig 2b).

### Splanchnic PIIIP assay

During surgery, serum s-PIIIP in IBD patients was 5.5 ± 2.6 μg/l. No significant differences were found when comparing CD and UC subgroups (5.4 ± 2.3 vs 5.7 ± 3.1 μg/l, respectively; p = ns). A positive gradient was found in IBD patients between splanchnic and peripheral serum concentrations of PIIIP (0.7 ± 1.9 μg/l). This gradient was confirmed when separately considering each disease, without significant differences between the two subgroups (CD 0.3 ± 1.3 vs UC 1.3 ± 2.6 μg/l; p = ns) (fig 2c).

### Other variables and PIIIP levels

No significant correlation was found between peripheral and splanchnic levels of PIIIP and the age of the patients and the estimated duration of the disease. Regarding the activity indices, the number of patients belonging to each class was not enough to perform a statistical analysis. Notwithstanding, for the TWI in UC patients a significant difference in PIIIP levels was found between mild and severe form of the disease (Table 3). Finally, no significant differences were found in PIIIP levels between patients treated with glucocorticoids compared with patients not receiving this treatment.

**Table 3:** Baseline p-PIIIP levels in UC patients

**TWI Activity Index**	**Mean ± SD**
**Mild**	7.05 ± 2.25
**Moderate**	3.9 ± 1.11
**Severe**	3.13 ± 1.57*

* p = 0.02 vs mild

## Discussion

CD and UC are chronic pathologies characterized by an early onset followed by sporadic episodes of acute symptoms during lifetime, debilitating the affected patients to perform their daily functions [24]. Until now controversial theories exist about the synthesis and degradation of PIIIP, its level on systemic circulation, and its deposition far for main target organ [12,13,15,25].

In the present study we have found that intestinal collagen metabolism in IBD patients was increased and that it is reflected in local and systemic circulation. Differently from some experiences [12,13], we have found that serum PIIIP levels in IBD patients was significantly higher if compared with healthy subjects. No significant differences were found in peripheral and splanchnic circulation between patients affected by UC and CD. We also found a positive gradient between serum s-PIIIP and p-PIIIP levels in IBD patients. This gradient was confirmed when considering serum s-PIIIP and p-PIIIP in UC and CD separately, even if the differences between the two subgroups were not statistically significant. In our experience no significant differences were found when considering the age of the patients, the duration of the disease, and the activity indices. This fact implies that serum PIIIP should not be considered a long-term marker of the disease, probably reflecting the short-term fluctuation in the activity phases of the remodeling processes.

When comparing the mild with the severe form of the disease, a significant difference in PIIIP levels was found only in patients affected by UC. This data will probably be confirmed when the number of patients enrolled in each disease-related activity categories is extended as presently in our series the majority of the patients were classified as severe.

The effect of glucocorticoids on collagen synthesis, collagenase, and collagen degradation has not yet fully been clarified [25]. In our study the cortisone therapy did not have influence on the PIIIP levels, but the number of patients was too small and it was not possible to speculate on this regard.

## Conclusions

In conclusion we provide evidence that collagen metabolism in IBD is reflected in the systemic and local circulation, without any differences between UC and CD, irrespective of the age of the patients and the duration of the disease. Therefore, this marker may give further information on the activity phases rather than on the entire history of the disease. Further data on the possible use of PIIIP as useful marker of choice for surgical option are attended from the follow-up at 6 and 12 months, which is still on-going [26].

## List of abbreviations

Inflammatory bowel diseases = IBD

Procollagen III propeptide = PIIIP

Peripheral Procollagen III propeptide = p-PIIIP

Splanchnic Procollagen III propeptide = s-PIIIP

Crohn's disease = CD

Ulcerative Colitis = UC

Crohn's Disease Activity Index = CDAI

Truelove-Witts index = TWI

## Competing interests

The author(s) declare that they have no competing interests.

## Authors' contributions

UC conception and design, interpretation of data, drafting the article

MDS conception and design, interpretation of data, drafting the article

ECA performed surgical operations, critical revision of the article, final approval of the version

BO patients' enrollement, blood samples collection

RP statistical analysis, interpretation of data, drafting the article

AP echocardiographic studies

GB radioimmunoassays

EO radioimmunoassays

SF histological examinations

FM interpretation of data, critical revision of the article, final approval of the version

MMC conception and design, interpretation of data, drafting the article

All Authors read and approved the final manuscript.

## Pre-publication history

The pre-publication history for this paper can be accessed here:


